# Disease patterns in juvenile dermatomyositis patients from Mumbai, India

**DOI:** 10.1186/1546-0096-9-S1-P53

**Published:** 2011-09-14

**Authors:** RP Khubchandani, D Mankad, PR Chickermane

**Affiliations:** 1 Department of Pediatrics,Jaslok Hospital and Research Centre, Mumbai, India

## Background

There is paucity of data regarding disease patterns and outcome in children with juvenile dermatomyositis from the Indian subcontinent.

## Aim

Retrospective assessment of disease characteristics, complications/ associations, treatment modalities used and outcome in an inceptional cohort of 20 children with juvenile dermatomyositis at a tertiary care centre in western India.

## Materials and methods

Chart review of 20 patients with juvenile dermatomyositis receiving treatment at a tertiary hospital in Mumbai from 1997 to 2010.

## Results

20 patients (male-12, female-8) were diagnosed as having definite or probable juvenile dermatomyositis based on the Bohan and Peter criteria.. The mean age at diagnosis was 7.02 years(range 3 to 15.25 years) and the mean duration of disease prior to diagnosis was 7.5 months (range 3 weeks-2 years).The most frequent symptoms at presentation were typical skin rash(100%), proximal muscle weakness(20/20-100%), fatigue(16/20-80%), fever(13/20-65%) and arthralgia(11/20-55%).Dysphagia was seen in 5/20(25%), cutaneous ulcers in 4/20(20%), calcinosis in 3/20(15%), lipodystrophy in 2/20 (10%) and pyogenic infections in 2/20 (10%). Hirsutism was seen in 3/20(15%) and vitiligo in 1/20(5%). One of the patients had associated insulin dependent diabetes mellitus and chronic hepatitis. None of the patients in the cohort presented with or developed cardiomyopathy. All patients presented with at least one of the four serum muscle enzyme levels elevated. Interestingly, a normal range of creatine kinase (CK.) was seen in 11/20 (55%) of the patients. Electromyography was done in 4 patients and muscle biopsy done to confirm the diagnosis in one patient. Magnetic resonance imaging of the thigh muscles was used to as an aid to diagnosis in 10/20 patients and was abnormal in all

Pulse steroids were used to induce remission in 10 patients and as a rescue for relapses in one patient. IVIG was used as rescue therapy in 2 children with good results. All patients received oral steroids and methotrexate as the standard maintanence treatment. Hydroxychloroquine was added in patients with dominant cutaneous manifestations. 4 children with inadequate response, ulcerative disease or hepatotoxicity received Azathioprine (4/4), cyclosporine (1/4), cyclophosphamide(1/4) as additional therapy. One child with extensive calcinosis is being treated with monthly IVIG, diltiazem and colchicine. The total follow-up period was 62.24 patient-years (range- 0.58 to 12.17 years). A monocyclic course was seen in 15/20(75%), relapsing course in 3/20 (15%) and a chronic progressive course in 2/20(10%).No mortality has been reported till date.

## Conclusions

There was a considerable delay at the primary care level for the diagnosis of juvenile dermatomyositis. A normal CPK, probably linked to this delay, often led the primary care pediatricians away from the diagnosis. Calcinosis, decreasingly reported in Western series is still seen here and may probably be linked to this delay.MRI has proven to be a useful tool in the diagnosis and has largely eliminated the need for a muscle biopsy.

Notwithstanding the delay in diagnosis, absent mortality, absence of cardio-pulmonary complications and a monocyclic course in 75% of our patients are at variance from several series from the West, which report severe disease.

